# Sex as a biological variable in human dental pulp stem cells: An exploratory epigenomic and transcriptomic comparison

**DOI:** 10.1016/j.reth.2026.101117

**Published:** 2026-04-21

**Authors:** Shuntaro Yamada, Kateřina Holomková, Niyaz Al-Sharabi, Tengyang Qiu, Hiroshi Egusa, Inge Fristad, Ana Angelova Volponi

**Affiliations:** a Center of Translational Oral Research, Department of Clinical Dentistry, University of Bergen, Bergen, Norway; b Centre for Craniofacial & Regenerative Biology, Faculty of Dentistry, Oral & Craniofacial Sciences, King's College London, London, UK; c Department of Histology and Embryology, Faculty of Medicine, Masaryk University, Brno, Czechia; d Institute of Animal Physiology and Genetics, Czech Academy of Sciences, Brno, Czechia; e Department of Mechanical Engineering, University College London, London, UK; f Developmental Biology and Cancer Research & Teaching Department, Great Ormond Street Institute of Child Health, University College London, London, UK; g Center for Advanced Stem Cell and Regenerative Research, Tohoku University Graduate School of Dentistry, Miyagi, Japan; h Division of Molecular & Regenerative Prosthodontics, Tohoku University Graduate School of Dentistry, Miyagi, Japan

**Keywords:** Dental stem cells, Mesenchymal stem cells, Biological sex, Transcriptome, Epigenetics, Regenerative medicine

## Abstract

**Objective:**

To determine whether baseline epigenomic and transcriptional profiles of human dental pulp stem cells (DPSCs) differ by sex, and to assess sex dependence of key markers related to growth and stemness.

**Methods:**

Primary DPSCs from nine young adult donors were isolated and cultured under standard growth conditions. Genome-wide DNA methylation was screened by Illumina Infinium Methylation EPIC v2.0. Bulk RNA sequencing was performed with poly A selected libraries on an Illumina NovaSeq X Plus sequencer. Additionally, a targeted RT-qPCR array covering canonical proliferation, stemness (multi- and pluri-potency, neural crest markers), MSC identity, and WNT and NOTCH signaling markers was tested.

**Results:**

Global differences between male- and female-derived samples were predominantly confined to the sex chromosomes. DNA methylation profiling revealed sex-dependent patterns, with mild autosomal hypermethylation in males and marked hypermethylation of the X chromosome in females. This pattern was accompanied by female-specific expression of XIST, supporting X-chromosome inactivation as a major contributor to the observed sex-chromosome signal. Autosomal methylation differences were generally modest (|Δβ| < 0.2). At the transcriptomic level, only a small fraction of genes (52 of 17,204) was differentially expressed, with most mapping to the sex chromosomes. Consistently, the RT-qPCR array indicated minimal differences between male and female DPSCs, with no consistent sex dependence across targets except for CCNE1.

**Conclusions:**

Under basal culture conditions, DNA methylation and transcriptional profiles in DPSCs are largely similar between sexes. The limited differential signal is associated predominantly with sex chromosomes, while autosomal effects are few and modest. These findings provide an initial molecular baseline and motivate larger and context specific studies.

## Highlights

• DNA methylation patterns in human dental pulp stem cells (DPSCs) are sex dependent, with autosomes showing mild hypermethylation in males and sex chromosomes hypermethylated in females.
• Differentially methylated loci (DMLs) across autosomes are modest in magnitude, with only a very small absolute methylation difference (|Δβ| < 0.2).
• Despite notable differences in DNA methylation patterns, transcriptional variation between male and female DPSCs is minimal and predominantly confined to sex-linked genes.
• Key markers associated with mesenchymal stem cell (MSC) identity, multipotency, and self-renewal show no sex-related differences at the transcriptional level.

## Graphical abstract

**Figure:**
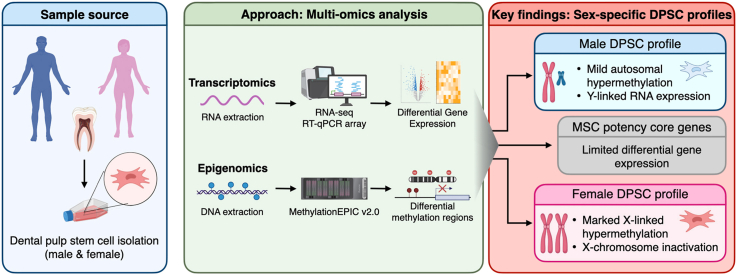

**Table:** Abbreviations

cDNA	Complementary deoxyribonucleic acid
CpG	Cytosine-phosphate-Guanine dinucleotide
Ct	Cycle threshold
DEGs	Differentially expressed genes
DMLs	Differentially methylated loci
DMEM	Dulbecco's Modified Eagle's Medium
DMSO	Dimethyl sulfoxide
DPSCs	Dental pulp stem cells
EPIC	Illumina Infinium MethylationEPIC BeadChip
FBS	Fetal bovine serum
FC	Fold change
FDR	False discovery rate
GRCh38/hg38	Genome Reference Consortium Human Build 38
lncRNA	Long non-coding RNA
log_2_FC	Log2 fold change
MSCs	Mesenchymal stem/stromal cells
mRNA	Messenger RNA
PCA	Principal component analysis
PCR	Polymerase chain reaction
PPI	Protein–protein interaction
qPCR	Quantitative polymerase chain reaction
RNA-seq	RNA sequencing
rlog	Regularized logarithm transformation
RT-qPCR	Reverse transcription quantitative polymerase chain reaction
STRING	Search Tool for the Retrieval of Interacting Genes/Proteins
TE	Tris–EDTA buffer
TSS200/TSS1500	CpG sites within 200 or 1500 bp upstream of transcription start site
XCI	X-chromosome inactivation

## 1 Introduction

Dental stem cells are a subset of mesenchymal stem/stromal cells (MSCs) widely recognized as a promising resource for regenerative medicine and dentistry, owing to their multipotency and rich secretome [1,2]. Among them, dental pulp stem cells (DPSCs), originally isolated from the dental pulp of permanent teeth, are particularly attractive because of their accessibility, non-invasive collection procedures, and rapid proliferation in culture [3]. DPSCs can differentiate into multiple mesenchymal lineages, including osteogenic, chondrogenic, and adipogenic cells, under defined conditions [4]. In addition to their differentiation potential, DPSCs secrete a broad range of trophic and immunomodulatory factors that contribute to angiogenesis, neuroprotection, and wound healing [5].

The clinical potential of DPSCs has been increasingly demonstrated in both preclinical and clinical contexts. To date, DPSCs have been tested in clinical trials and case reports for oral and craniofacial regeneration, including pulp-dentin complex regeneration, periodontal regeneration, and craniofacial bone regeneration [6]. Their application has further extended to systemic conditions, such as graft-versus-host disease, acute ischemic stroke, Huntington's disease, and COVID-19-associated complications. These studies highlight the versatility of DPSCs as a cell source for regenerative medicine, extending beyond dentistry [7]. Reflecting this translational potential, public and private biobanking initiatives have emerged globally to enable the long-term preservation of patient-derived DPSCs, aiming to secure personalized stem cell sources for future therapeutic use [7].

In parallel with the growing interest in clinical translation, increasing attention has been directed towards sex as a biological variable in biomedical research. Although the importance of sex- and gender-based analysis is now widely recognized, most fundamental and translational studies have historically been conducted without considering donor sex, under the assumption that biological processes were largely equivalent between males and females. Even today, only a limited number of studies systematically incorporate or report sex-related analyses. One reason could be that cell sources obtained from commercial providers or biobanks often lack donor sex information, either because it is not collected, or because disclosure is restricted by privacy and regulatory considerations. In addition, investigators may omit reporting sex due to perceived irrelevance, small sample sizes, or concerns that stratification will reduce statistical power. Nevertheless, accumulating evidence shows that sex-based differences can significantly influence disease susceptibility, treatment responses, and regenerative potential [8]. In MSC biology specifically, donor sex has been suggested to affect MSC characteristics, including proliferation rates [9], differentiation bias [10], and immunomodulatory activity [11]. Notably, such observations have been reported primarily in MSCs derived from bone marrow, umbilical cord, and adipose tissue, and have been documented not only in human studies but also across several animal models, including mouse, rat, monkey, and pig [8]. Such differences are thought to arise from a combination of genetic factors, hormonal influences, and epigenetic regulation [11].

Despite this growing recognition, systematic investigations of sex-related differences in dental stem cells remain scarce. An early study reported potential differences in the proliferation and osteogenic differentiation capacity of human DPSCs between male and female donors, which were suggested to correlate with telomere length [12]. In contrast, another study comparing 18 male and 32 female samples did not find any significant sex-related differences in proliferation [13]. Beyond these limited reports on proliferation and osteogenic differentiation, no additional evidence, nor even exploratory data, exists regarding sex-specific differences in DPSCs. This represents a critical knowledge gap, as understanding whether male and female DPSCs differ at the molecular level is essential for ensuring reproducibility and optimizing donor selection. To address this, we compared the epigenomic and transcriptional profiles of DPSCs derived from male and female donors using genome-wide DNA methylation screening and bulk-RNA sequencing complemented by a targeted gene expression array. To our knowledge, this is the first study to apply high-throughput multi-layered analyses to investigate sex-related differences in human primary DPSCs.

## 2 Materials & methods

### 2.1 DPSC Isolation and expansion

The use of human samples was approved by the Regional Committee for Medical and Health Research Ethics in Norway (2009/610/REK vest). Dental pulp tissue was obtained at the affiliated clinic of the Department of Clinical Dentistry, University of Bergen, from premolars extracted from ten healthy donors (five male, five female), aged 18–24 years, following informed consent. All teeth had fully developed apices, were free of caries, and showed no clinical or radiographic signs of inflammation or pathology in the pulp or periodontal tissues.

Pulp tissue was enzymatically digested with collagenase type I (4 mg/mL) and dispase (2 mg/mL) for 1 h at 37 °C, and the resulting cell suspension was plated into culture flasks. DPSCs were expanded in growth medium consisting of Dulbecco's Modified Eagle's Medium (DMEM; 10566016, Gibco, USA) supplemented with 10% fetal bovine serum (FBS; 10270-106, Gibco, USA) and 1% penicillin/streptomycin (SV30010, HyClone, USA). Cultures were maintained at 37 °C in a humidified incubator with 5% CO_2_. DPSCs from one male donor yielded insufficient cell numbers and were therefore excluded from subsequent analyses. At passage 1, cells were cryopreserved in a freezing medium composed of 10% dimethyl sulfoxide (DMSO), 20% FBS, and 70% DMEM, and stored in liquid nitrogen until further use. For this study, DPSCs were thawed, cultured to ∼80% confluency, and then expanded to passage 2. Cells from nine donors were analyzed, with each donor evaluated independently. DPSCs from all the donors were preliminarily assessed for and met the minimum MSC criteria in accordance with the position paper from the International Society for Cell & Gene Therapy, including spindle-shape morphology, multi-lineage differentiation, and MSC marker expression (data not shown) [14].

### 2.2 Sample collection and nucleic acid extraction

DPSCs at 80% confluency were washed in PBS once, scraped, collected into 1.5-mL Eppendorf tubes, and immediately snap-frozen in liquid nitrogen. This process was completed within 7-8 min to preserve the physiological transcriptional profile in culture.

Double strand DNA was extracted using a DNeasy Blood & Tissue Kit (69504, Qiagen, Germany) in accordance with the manufacturer's protocol. Purified DNA was eluted in TE buffer and stored at −20 °C until use.

Total RNA was then extracted using the Maxwell® 16 Cell LEV Total RNA Purification Kit (AS1280, Promega, USA) in an automated Maxwell® processing system according to the manufacturer's instructions. Purified RNA was eluted in nuclease-free water and stored at −80 °C until use.

### 2.3 Genome-wide DNA methylation profiling

The genome-wide DNA methylation profile was screened by using the Illumina Infinium MethylationEPIC V2 BeadChip array (935K), with the Epigenomic Services from Diagenode (G0209006), covering over 900,000 human CpG positions throughout the genome with single nucleotide resolution. After quality control (QC) and filtering, 858,985 probes were included for analysis.

### 2.4 Bulk RNA sequencing

Bulk RNA sequencing (RNA-seq) was performed by Novogene Ltd. (Cambridge, UK). Messenger RNA (mRNA) libraries were prepared using poly(A) enrichment. Briefly, total RNA was enriched for polyadenylated transcripts, which were subsequently fragmented and reverse-transcribed to generate cDNA. Following end repair, A-tailing, adaptor ligation, and PCR amplification, the final libraries were quantified and assessed for quality. Sequencing was performed on the NovaSeq X Plus platform (Illumina, USA) in paired-end mode, yielding approximately 6 Gb of raw data per sample.

### 2.5 Reverse transcription quantitative polymerase chain reaction (RT-qPCR)

Reverse transcription was performed using the High-Capacity cDNA Reverse Transcription Kit (4368814, Applied Biosystems, USA). RT-qPCR was carried out on a StepOne™ Real-Time PCR System (Applied Biosystems) with custom-designed array panel, covering 44 unique genes and 3 endogenous control genes (4413257, Applied Biosystems; Table S1). The genes include key markers for cell proliferation (cell cycle regulators), potency (multi- and pluri-potency markers, neural crest markers), and MSC identity. Amplification conditions consisted of an initial denaturation at 95 °C for 20 s, followed by 40 cycles of 95 °C for 2 s and 60 °C for 20 s. Reaction specificity was confirmed by amplification curve analysis, and relative gene expression levels were calculated using the ΔΔCT method [15], normalized to the average of housekeeping genes (*GAPDH, ACTB, HPRT1*).

### 2.6 Bioinformatics and statistics

For DNA methylation analysis, IDAT files were processed in R (v4.4.0) using the minfi and SeSAMe packages. Background correction and dye-bias normalization were performed using the noob method. Probes with detection p > 0.01, cross-reactive probes, or those overlapping known single-nucleotide polymorphisms were excluded from analysis. QC and data filtering was conducted by Diagenode while subsequent analysis was conducted by the authors. Beta values (β), representing the proportion of methylated signal intensity, were calculated for each CpG site. Δβ (i.e. difference in methylation levels) was calculated as:

Δβ=β(male)−β(female)

P-values were adjusted for multiple testing using the Benjamini–Hochberg false discovery rate (FDR). CpG sites with adjusted p-value < 0.05 and absolute methylation difference (|Δβ|) > 0.2 were defined as differentially methylated loci (DMLs). Sites showing higher methylation in males were classified as hypermethylated, and those with lower methylation as hypomethylated. Probes were annotated to their corresponding genes and genomic features using the EPIC-8v2-0_A1 manifest from Illumina. CpG sites were grouped by chromosomal location (autosomes, X, and Y chromosomes) to evaluate sex-specific methylation patterns. Visualization (volcano plot, density plot, Manhattan plot, and bubble plot) was performed in R using ggplot2, qqman, and patchwork. GraphPad Prism 10 was used to generate violin plots.

For RNA-seq data analysis, raw sequencing files (.*fastq*) were processed using the open-source Galaxy platform (EU server). Read quality was assessed with FastQC, and high-quality reads were aligned to the human reference genome (GRCh38/hg38) using HISAT2. AnnotateMyIDs was used to annotate gene ID. Gene-level counts were generated with featureCounts to produce count matrices. Differential expression analysis was performed with DESeq2, and genes with an adjusted p-value (Benjamini–Hochberg) < 0.05 and an absolute log_2_ fold change (FC) > 1 were considered significantly differentially expressed. Normalized counts and regularized log-transformed (rlog) data were used for visualization on SR plot [16], including principal component analysis (PCA), correlation plot, heatmaps, and chromosome alignment, and on R for a MA plot. Protein–protein interaction (PPI) analysis was performed on protein-coding, differentially expressed genes (DEGs) (non-coding/pseudogenes excluded) using STRING (*Homo sapiens*; high-confidence score ≥0.7).

For integrative multi-omics analysis, RNAseq differential expression data (log_2_FC) and EPIC array data (Δβ) were integrated to examine the relationship between gene expression and promoter methylation. CpG probes annotated to promoter-proximal regions (TSS200, TSS1500, and 1st exon) were averaged to obtain a single mean Δβ value per gene. Genes common to both datasets were analyzed in R, and the association between promoter methylation and expression was evaluated using Spearman's rank correlation. Visualization was performed using the ggplot2 package within the tidyverse framework, displaying RNAseq log_2_FC on the x-axis and mean promoter Δβ on the y-axis. A linear regression line with 95% confidence interval was overlaid to illustrate the overall trend. The R package circlize was used for chord diagram visualization.

For RT-qPCR, comparisons between male- and female-derived DPSCs were performed using an unpaired Welch's *t*-test, which does not assume equal variance.

All data are represented as mean ± S.D., unless mentioned otherwise. A (adjusted) p-value of <0.05 was considered statistically significant.

## 3 Results

### 3.1 Genome-wide DNA methylation profile of male- and female-derived DPSCs

**Fig. 1:**
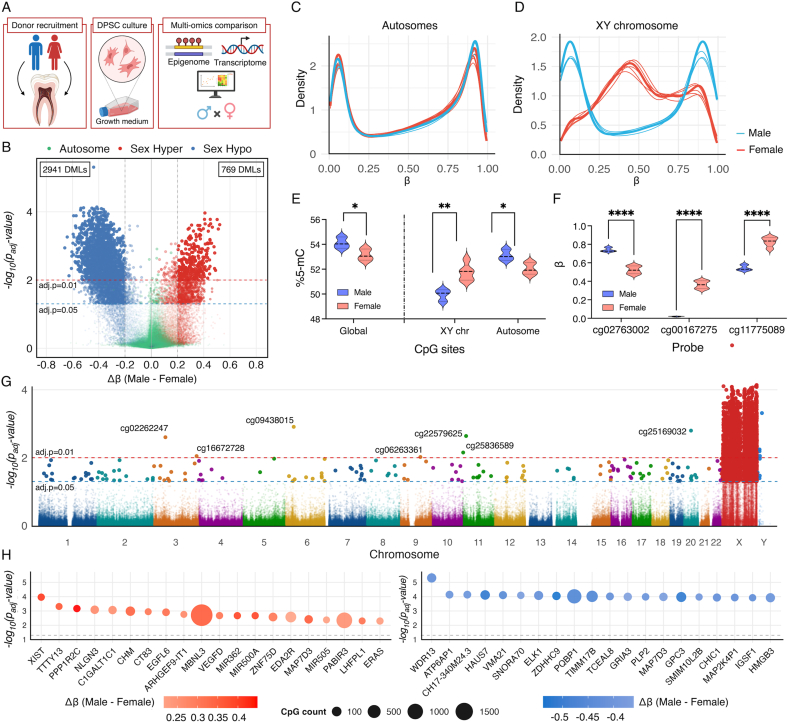
**Sex-associated genome-wide DNA methylation profiles in human dental pulp stem cells (DPSCs).** (A) Schematic illustration of study design. (B) Volcano plot showing differentially methylated CpG sites between male and female DPSCs. Red and blue indicate hypermethylated and hypomethylated sites in males, respectively, while green points represent autosomal CpGs. (C) Density distributions of β-values across all autosomal and (D) sex chromosomal CpG sites, stratified by sex. (E) Violin plots showing global, sex chromosomal (XY), and autosomal 5-methylcytosine (%5-mC) levels between male and female DPSCs. (F) Differentially methylated CpG sites (|Δβ (male – female)| > 0.2; adjusted p value < 0.05) on autosomes. (G) Manhattan plot displaying genome-wide distribution of DMLs. Each dot represents one CpG site plotted against chromosomal position. Red and blue dashed lines indicate the significance thresholds for BH-adjusted p-value (padj.) <0.01 and < 0.05, respectively. (H) Gene-level summary of top 20 hypermethylated (left, red) and hypomethylated (right, blue) genes within promoter-proximal regions in males relative to females. Circle size represents CpG count per gene, and color intensity corresponds to Δβ magnitude. ∗∗∗∗ padj. < 0.0001, ∗∗ padj. < 0.01, ∗ padj. < 0.05.

To investigate sex-specific DNA methylation patterns in human DPSCs, we performed genome-wide DNA methylation profiling. Sex accounted for a substantial proportion of the observed methylation variance, with the most pronounced differences localized to the sex chromosomes and more modest yet consistent shifts across autosomes (Fig. 1A–C). Male-derived samples exhibited a trend toward hypomethylation across CpG sites on the sex chromosomes, consistent with the higher methylation of the inactive X chromosome in females, whereas autosomal regions showed a slight tendency toward hypermethylation in males (Fig. 1D and E, Table 1, Table S2). Nevertheless, these differences were relatively modest (|Δβ| < 0.2), and only three CpG sites were identified as DMLs between male and female DPSCs (Fig. 1F).

**Table 1:** Distribution of hypermethylated and hypomethylated CpG sites across chromosome groups.

P-adj. < 0.05; |Δβ| > 0.2	Autosomes	Sex chromosomes	
		X	Y
Hypermethylated in males	1	761	7
Hypomethylated in males	2	2939	0
Not significant	835,802	19,402	71
% DML per chromosome group	<0.01%	16.0%	9.0%

Genome-wide association across chromosomes revealed numerous autosomal loci but a striking enrichment of highly significant sites on the X chromosome (Fig. 1G). Several probes, including cg02262247, cg09438015, cg16672728, cg06263361, cg22579625, cg25836589, and cg25169032, presented discrete hotspots of sex-biased methylation despite exhibiting only modest effect sizes (Δβ < 0.2). Gene-level aggregation further demonstrated that the top 20 DMLs encompassing both hypermethylated and hypomethylated CpG sites within promoter-associated regions (TSS200/TSS1500) were predominantly located on the sex chromosomes, whereas no autosomal loci reached comparable significance (Fig. 1H).

### 3.2 Differential transcriptomic profiles of male- and female-derived DPSCs

**Fig. 2:**
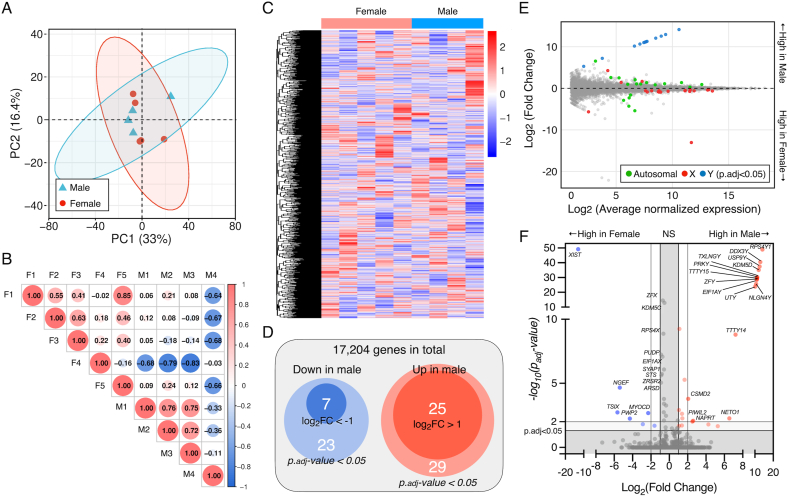
**Bulk RNA-seq of human DPSCs from male and female donors.** (A) Principal component analysis of rlog-normalized counts for the top 500 most variable genes with concentration eclipses showing within-group dispersion. Each point represents one donor. (B) Pairwise sample–sample correlation matrix between male (M) and female (F) DPSCs. Colors show Pearson correlation coefficients. (C) Unsupervised hierarchical clustering heatmap of all expressed genes using Euclidean distance and complete linkage. (D) Summary of differentially expressed genes (DEGs) in male DPSCs compared to female DPSCs. Outer circles show totals at adjusted p value < 0.05, and inner circles show the subset with |log_2_FC| > 1. (E) MA plot showing sex-biased gene expression in DPSCs. Statistically significant DEGs (adjusted p < 0.05) are marked in green, blue, and red for autosomal, X-linked, and Y-linked genes, respectively. (F) Volcano plot of RNA-seq data with represented genes labelled.

Next, the transcriptomic profile was characterized by bulk-RNAseq. PCA analysis using a top 500 variance revealed that the major source of variance in the dataset was inter-donor heterogeneity rather than sex, as male and female samples showed broad overlap along the principal components (Fig. 2A). Similarly, pairwise Pearson correlation analysis confirmed high similarity across all donors, with no clear stratification by sex (Fig. 2B). Unsupervised hierarchical clustering of global expression profiles further supported this observation, with samples clustering in a mixed fashion independent of donor sex (Fig. 2C). Nevertheless, differential expression analysis identified a small subset of DEGs (i.e. adjusted p-value < 0.05), including 29 upregulated and 23 downregulated transcripts, among 17,204 tested genes, in male DPSCs (Fig. 2D). The DEGs with substantial magnitude were predominantly sex chromosomal, particularly Y-linked, while autosomal changes were modest, highlighting these sex-associated expression patterns within the broader transcriptome (Fig. 2E and F).

### 3.3 Chromosomal distribution of differentially expressed genes

**Fig. 3:**
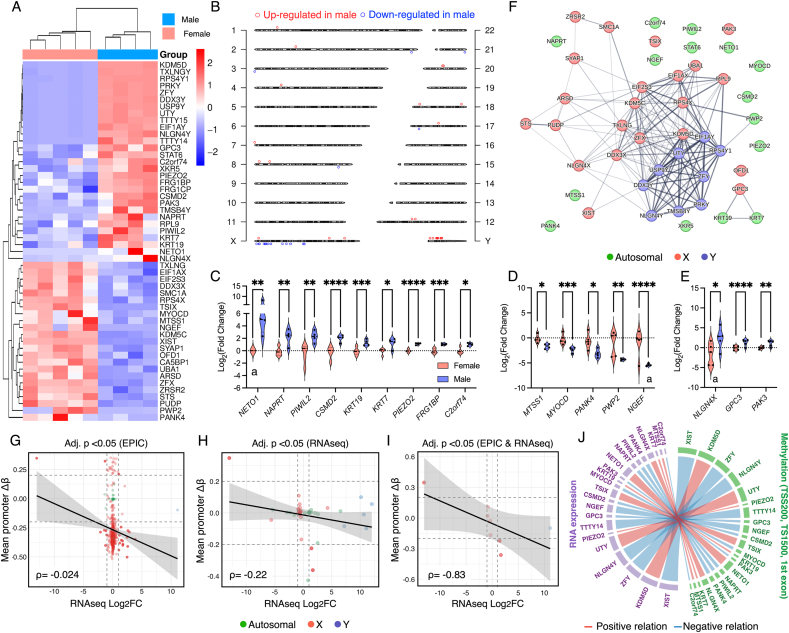
**Chromosomal distribution of Differentially Expressed Genes (DEGs).** (A) Heatmap of all DEGs (adjusted p-value < 0.05) using rlog-normalized counts. Hierarchical clustering of genes and samples was performed using Euclidean distance and complete-linkage agglomeration. (B) Ideogram mapping of DEGs. Red and blue circles denote up- and down-regulated genes in male DPSCs, respectively. (C, D) Violin plots of autosomal genes upregulated and downregulated in male DPSCs with |log_2_FC| > 1 and adjusted p-value < 0.05. (D) Violin plots of X-chromosomal genes upregulated in males. Points represent individual donors (E) Protein–protein interaction network built from protein-coding DEGs in STRING at medium confidence score 0.40. The network contains 46 nodes and 163 edges, with an average node degree of 7.1 and an average local clustering coefficient of 0.48. Connectivity is greater than expected by chance, with 17 edges expected at random and a PPI enrichment (p-value < 1.0 × 10^−16^). Node color encodes chromosomal location. (G-I) Integrated methylation–expression correlation plots of genes (male vs female) with adjusted p-value threshold for EPIC array or/and RNAseq, showing negative relation between promoter methylation and RNA expression. (J) Circos plot illustrating cross-omics correlations. Chords were colored according to the direction of association (red for positive and blue for negative relationships), and their width was scaled by the combined magnitude of |log_2_FC| and |Δβ|. ∗ padj.<0.05, ∗∗ padj.<0.01, ∗∗∗ padj.<0.001, ∗∗∗∗ padj.<0.0001. ^a^ normalized count <5.

Restricting the analysis to genes with adjusted p-value < 0.05 (incl. genes with |log_2_FC| < 1), unsupervised clustering separated the two donor groups more clearly than the whole-transcriptome view (Fig. 3A). Mapping DEGs to their genomic locations revealed a marked concentration on the sex chromosomes, with only a small number distributed across autosomes (Fig. 3B). Among upregulated DEGs, 16/29 were on sex chromosomes (X: 3; Y: 13) and 13/29 on autosomes; among downregulated DEGs, 18/23 were on the X chromosome and 5/23 on autosomes. Overall, these correspond to ∼0.17% (upregulated) and ∼0.13% (downregulated) of all detected genes, indicating that the limited differential signal is largely attributable to loci on the sex chromosomes, with minimal autosomal contribution (Table 2, Table S3).

**Table 2:** Differentially expressed genes (DEGs) by chromosomal group among 17,204 detected genes.

DEGs (adj. p < 0.05)	Autosomes	Sex chromosomes		Total DEGs	% DEGs
		X	Y		
Upregulated in males	13	3	13	29	0.17%
Downregulated in males	5	18	0	23	0.13%
Total DEGs	18	21	13	52	0.30%
% DEGs	0.11%	0.12%	0.076%	0.30%	

For further analysis, DEGs with a FC threashold (i.e. adjusted p-value < 0.05, |log_2_FC| > 1) were stratified by chromosomal origin. Nine autosomal genes were upregulated in male DPSCs (Fig. 3C), including *NETO1*(chr18; log_2_FC = 6.55; adjusted p = 0.0053), *NAPRT* (chr7; log_2_FC = 2.60; adjusted p = 0.0084), and *PIWIL2* (chr8; log_2_FC = 2.51; adjusted p = 0.0095). On the contrary, five autosomal genes showed lower expression in male DPSCs (Fig. 3D), including *NGEF* (chr2; log_2_FC = −5.39; adjusted p = 2.14 × 10^−5^), *PWP2* (chr21; log_2_FC = −4.29; adjusted p = 0.00564), and *MYOCD* (chr17; log_2_FC = −2.30; adjusted p = 0.00209). X-linked transcripts with higher expression in males were also observed (Fig. 3E), represented by *NLGN4X* (log_2_FC = 4.26; adjusted p = 0.0161), *GPC3* (log_2_FC = 1.67; adjusted p = 5.27 × 10^−6^), and *PAK3* (log_2_FC = 1.43; adjusted p = 0.00536). Y-chromosomal transcripts were also upregulated as expected, comprising predominantly X-degenerate, single-copy protein-coding genes (e.g., *RPS4Y1*, *DDX3Y*, *USP9Y*, *KDM5D*, *EIF1AY*, *NLGN4Y*, *UTY*, *ZFY*, *TMSB4Y*), alongside Y-linked pseudogenes (*PRKY*, *TXLNGY*) and lncRNAs (*TTTY14*, *TTTY15*). The direction of change was consistent across donors, with limited overlap between groups. A protein–protein interaction network constructed from protein-coding DEGs revealed a dense module of sex-chromosome genes including X–Y homolog pairs (e.g., RPS4X/RPS4Y1, EIF1AX/EIF1AY, KDM5C/KDM5D), indicating coordinated connectivity among sex-linked transcripts (Fig. 3F). In contrast, autosomal DEGs showed sparse connectivity and appeared largely as peripheral or isolated nodes, suggesting limited functional interaction among the genes.

Integrated multi-omics analysis revealed that methylation of CpG sites within promoter and gene-proximal regions (TSS200, TSS1500, and first exon) showed a weak negative trend with RNA expression (Spearman's rank correlation rho = −0.024 when genes were filtered by adjusted p-value < 0.05 for differential methylation; rho = −0.22 when filtered by adjusted p-value < 0.05 for differential expression), without reaching statistical significance (Fig. 3G and H). In contrast, genes overlapping between DML and DEG exhibited a strong and significant inverse correlation (r = −0.83, p = 0.0101) (Fig. 3I and J).

### 3.4 Targeted RT-qPCR array showing minimal sex-dependent differences

**Fig. 4:**
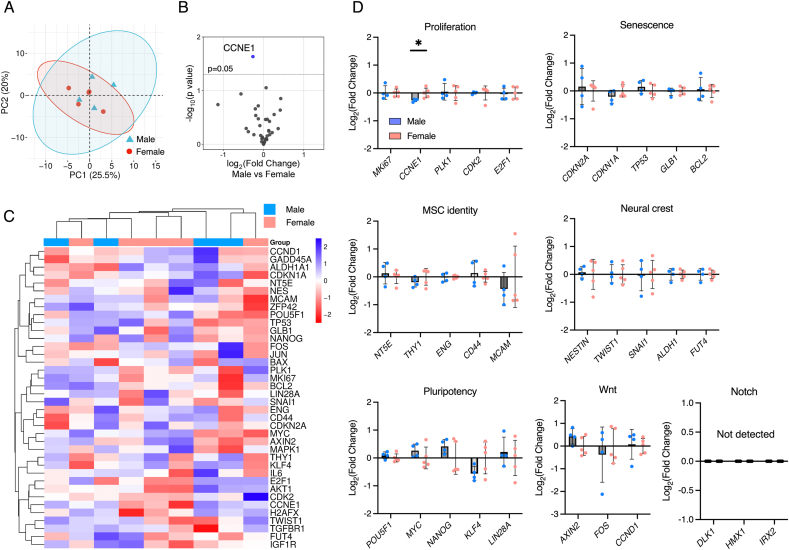
**Targeted RT-qPCR array of key functional markers in male and female DPSCs** (A) Principal component analysis of ΔCt-normalized expression across 44 targets; each point represents one donor. Concentration ellipses depict within-group dispersion. (B) Volcano plot showing nominal differences between male and female DPSCs. Down regulation in male is indicated in Blue. (C) Heatmap of z-scored ΔCt level with hierarchical clustering of genes and samples using Euclidean distance and complete linkage; top bar indicates donor sex. (D) Category summaries of marker expression for proliferation, senescence, MSC identity, neural crest, pluripotency, and WNT and NOTCH-related markers; bars show mean ± SD and points show individual donors. ∗p < 0.05 (Welch's *t*-test).

A targeted RT-qPCR array was designed specifically to test whether canonical stemness and growth-related genes display sex-dependent expression. PCA showed substantial overlap between male and female samples with no global separation (Fig. 4A). The volcano plot indicated only a nominal effect for *CCNE1* and otherwise non-significant differences (Fig. 4B). An unsupervised heatmap of z-scored ΔCt values displayed intermingled clustering without sex-specific segregation (Fig. 4C). Category summaries (i.e. mean log_2_ FC) were centered near zero for proliferation markers *(MKI67, PLK1, CDK2, E2F1*) except for *CCNE1* (p = 0.0249), senescence (*CDKN2A, CDKN1A, TP53, GLB1, BCL2*), MSC identity (*NT5E, THY1, ENG, CD44, MCAM*), neural crest (*NES, TWIST1, SNAI1, ALDH1A1, FUT4*), pluripotency (*POU5F1, MYC, NANOG, KLF4, LIN28A*), and WNT-related genes (*AXIN2, FOS, CCND1*), indicating an absence of consistent sex bias (Fig. 4D). NOTCH-panel targets in this assay (*DLK1, HMX1, IRX2*) were not detected under the basal culture conditions.

## 4 Discussion

There is growing recognition that biological sex can influence cellular phenotypes across biomedicine, including dentistry, and funding agencies increasingly expect sex to be considered in study design and reporting [17]. Comparative work in MSCs from bone marrow, adipose tissue, and umbilical cord has reported sex-associated differences in proliferation, differentiation bias, senescence, and immunomodulatory activity, although effects are context dependent and not universal [8,11]. By contrast, gender dimension and biological sex in dental stem cells have been sparsely explored, with available evidence limited to a few functional observations with mixed conclusions. Consistent with this gap, the recent single-cell human dental atlas utilized pooled donors of mixed sex and did not perform sex-stratified analyses [2]. To our knowledge, no prior study has provided a paired genome-wide DNA methylation and transcriptome-wide comparison of male versus female human primary DPSCs. The present work therefore offers the first high-throughput, exploratory views of potential sex-associated epigenomic and transcriptional profiles in DPSCs and establishes a molecular baseline as a reference to encourage future, larger and mechanistically oriented investigations.

Dental pulp tissue is well recognized as sex dependent. Cytogenetic studies show that sex chromatin markers in pulpal cells, including Barr bodies and Y bodies, can reliably indicate biological sex, which supports intrinsic anatomical differences within pulp tissue [18]. Physiological and functional evidence also supports sexual dimorphism. In *ex vivo* human dental pulp, serotonin preferentially enhanced capsaicin-evoked release of calcitonin gene related peptide in samples from women compared with men, indicating sex dependent trigeminal nociceptor signaling relevant to pain processing [19]. In preclinical models, central sensory processing after tooth pulp injury differs by sex, with distinct patterns of thalamic neuronal remodeling observed in males and females [20]. Clinically, symptomatic pulpitis shows greater upregulation of purinergic signaling components such as P2X3 and CD39 in women than in men, consistent with sex linked differences in nociception [21].

Despite well documented sex differences in pulp tissue biology, baseline epigenomic and transcriptional dimorphism in DPSCs appeared limited. In this exploratory study, only a small proportion of genes met the significance threshold after correction for multiple testing, indicating modest effect sizes and a signal largely confined to the sex chromosomes. Autosomal differences were rare and small in magnitude. At the DNA methylation level, most DMLs were located on the sex chromosomes, exhibiting a consistent trend of hypomethylation in male-derived DPSCs. In contrast, differences in autosomal CpG methylation were minimal, with only three CpG sites exceeding both the adjusted significance threshold (p-adj. < 0.05) and the effect size criterion (Δβ > 0.2). At the transcriptome level, approximately 0.3% of tested genes (52 of 17,204) met the adjusted significance threshold, with about 0.19% mapping to the sex chromosomes and roughly 0.11% on autosomes. This pattern indicates that the limited differential expression observed is driven predominantly, though not exclusively, by sex chromosome loci, whereas autosomal differences are few and modest in magnitude.

Importantly, the observed methylation asymmetries did not translate into large-scale transcriptional divergence. Differential expression remained modest and was largely confined to sex-chromosome genes, suggesting that epigenetic compensation mechanisms, such as X-chromosome inactivation (XCI) and dosage compensation, maintain overall transcriptional balance between male and female DPSCs while preserving locus-specific regulatory differences [22]. This is exemplified by the low promoter methylation and high expression of XIST, a master regulator of XCI, robustly expressed in female-derived DPSCs but silenced in males. Many other X-linked genes appeared transcriptionally buffered despite methylation differences, reflecting the nuanced interplay between methylation, chromatin accessibility, and transcriptional control [23]. Such subtle, sex-dependent epigenetic states may modulate chromatin structure and differentiation kinetics rather than basal gene expression, contributing to minor but reproducible phenotypic variations in DPSC growth or lineage potential [24,25].

The limited transcriptional changes between male and female-derived DPSCs observed in the present study well align with previous reports on sex-dependent differences in other types of human MSCs such as adipose tissue-derived [26], bone marrow-derived [8] and umbilical cord-derived MSCs [27]. Noteworthily, large cross-tissue studies that catalogue sex-biased expression in humans, for example Genotype-Tissue Expression (GTEx) [28] and the multi tissue analysis [29], did not cover dental tissues. Moreover, these did not identify the autosomal DEGs observed in the present study sex-biased in any analyzed tissues. This suggests that the autosomal differences observed in DPSCs may be tissue specific, subtle, or context dependent rather than universally sex linked. A meta-analysis across more than two thousand five hundred human samples found the largest sex differences in brain and heart with far fewer in other tissues, supporting the notion that strong sex biased expression is not expected in DPSCs [30]. Similarly, the targeted RT-qPCR array showed minimal differences between sexes across key markers that regulate cell growth and stemness in DPSCs. Only *CCNE1*, which encodes cyclin E1 and acts at the G1 to S phase transition, showed a nominal sex related difference with higher expression in males. Evidence from other tissues is limited with only a few cancer studies reporting sex-biased expression (e.g., lung) [31]. All other assayed genes showed no sex dependence, consistent with RNA-seq data, supporting a prior report of comparable DPSC functionality in males and females [13]. Taken together, baseline autosomal sex differences in DPSCs are likely modest and tissue specific rather than widespread across the transcriptome.

This study has limitations that guide interpretation while preserving its value. Firstly, due to modest sample size, the conclusion of the study remains exploratory rather than conclusive. To ensure robustness, we applied stringent thresholds for both DNA methylation (|Δβ| > 0.2) and transcriptomic analyses (|log_2_FC| > 1) in addition to adjusted p-value <0.05, which may have prevented the detection of small yet biologically relevant sex-associated differences. As a result, our analyses are primarily sensitive to relatively large differences between sexes, and more subtle effects may remain undetected, making the risk of type II errors non-negligible. Therefore, non-significant findings in this dataset should be interpreted as an absence of evidence for strong baseline dimorphism rather than evidence for complete equivalence between male- and female-derived DPSCs. Similarly, donor-to-donor variation beyond sex may obscure subtle sex-associated differences. For example, previous work has shown that age strongly influence the proliferative and differentiation potential of dental pulp cells rather than sex [32]. While our study focused on young adult donors to minimize age-related effects, donor-to-donor variation remains a significant source of heterogeneity [33,34]. Additionally, the present study utilized early passage for the analysis. Because primary DPSC cultures are intrinsically heterogeneous [2], this heterogeneity may dilute subtle sex-associated signals in bulk profiling. However, we view it as clinically relevant rather than a drawback, since DPSC/MSC products are typically administered as heterogeneous populations and such diversity contribute to improved regenerative potency [7,35]. We therefore prioritized early passages to capture a baseline molecular state that is closest to the clinically used manufacturing window and least influenced by culture-driven selection, subpopulation enrichment, and progressive homogenization during expansion [36]. Lastly, DPSCs were examined under the standard growth conditions, which captures a steady state. However, responses to stimuli or to different culture conditions such as other medium composition, inflammatory priming, hypoxia, or lineage induction may differ between sexes [37,38]. Further studies including diverse cohort, followed by meta-analysis, are needed to strengthen the generalizability of the baseline transcriptomic findings and consequently to clarify gender dimension in DPSCs.

## 5 Conclusion

This study provides the first genome-wide DNA methylation and transcriptome-wide comparison of male- and female-derived human DPSCs. Despite well-documented sex differences at pulp tissues and physiological levels, baseline epigenomic and transcriptional dimorphism in DPSCs was modest and largely confined to the sex chromosomes, with minimal autosomal variation. The lack of concordance between X-linked DNA methylation and gene expression likely reflects mechanisms of X-chromosome inactivation and dosage compensation in females. These findings suggest that, under steady-state conditions, sex exerts limited influence on the intrinsic molecular phenotype of DPSCs, although 1) context-dependent differences may emerge under inflammatory, hypoxic, or differentiation-inducing conditions, and 2) a larger cohort may reveal subtle yet phenotypically meaningful effects that may have been masked by the modest sample size in the present study. Accordingly, this study is best viewed as an exploratory reference dataset that defines the magnitude and distribution of sex-associated molecular differences in DPSCs under standard culture conditions. Future studies incorporating larger, age-balanced cohorts together with functional assays will be essential to validate whether subtle sex-associated signatures translate into clinically or phenotypically relevant effects.

## Declaration of generative AI in scientific writing

During the preparation of this work the author(s) used Microsoft Copilot in accordance with the institutional guidelines at University of Bergen to improve English language and readability. After using this tool, the authors reviewed and edited the content as needed and take full responsibility for the content of the published article.

## Funding

This work was supported in part by the Research Council of Norway (Småforsk grant) to I.F., research funds and Momentum stipend from 10.13039/501100005036University of Bergen to S.Y. and by the European Union Erasmus + program and Brno City Municipality to K.H.

## Declaration of competing interest

The authors declare the following financial interests/personal relationships which may be considered as potential competing interestsShuntaro Yamada reports financial support was provided by University of Bergen. Inge Fristad reports financial support was provided by Research Council of Norway. Katerina Holomkova reports financial support was provided by European Commission and Brno City Municipality. If there are other authors, they declare that they have no known competing financial interests or personal relationships that could have appeared to influence the work reported in this paper.
